# Clinical diagnosis and treatment experience of 21 case report of isolated sphenoid sinus lesions

**DOI:** 10.1097/MD.0000000000041812

**Published:** 2025-03-21

**Authors:** Ziwei Yu, Yanli Yu, Xudong Chen, Yaowen Wang, Peng Cheng

**Affiliations:** a Department of Otolaryngology, Head and Neck Surgery, The First Affiliated Hospital of Ningbo University, Ningbo, Zhejiang, China.

**Keywords:** cerebrospinal fluid rhinorrhea, inverted papilloma, isolated lesions of the sphenoid sinus, sphenoid sinus

## Abstract

**Rationale:**

With the rapid development of medical science and technology, imaging has become an important part of the diagnosis of diseases, which can provide important information about the morphology and scope of the lesion and its relationship with the surrounding tissues. However, due to the fact that isolated pterygoid sinus lesions often have atypical clinical symptoms, and the location of the lesion is deep and the anatomical structure is complex, its diagnosis still faces one of the important challenges, and the misdiagnosis rate is high. The aim of this study was to explore the clinical symptoms, imaging manifestations, and postoperative pathological features of isolated pterygoid sinus lesions, and to summarize the key points of their diagnosis through systematic retrospective analysis, with a view to providing references for clinicians, reducing the misdiagnosis rate of this disease, and improving the level of diagnosis and treatment.

**Patient concerns:**

A retrospective analysis was conducted on the clinical data of 21 patients with isolated sphenoid sinus lesions diagnosed and treated in the ENT department of our hospital from January 2021 to January 2023. Among them, there were 12 cases of headache, 1 case of eye symptoms, 10 cases of nasal symptoms, and 1 case of facial symptoms upon admission.

**Diagnoses:**

Among the 21 cases, there were 10 cases of fungal sphenoid sinus inflammation, 3 cases of sphenoid sinus cyst, 4 cases of chronic sphenoid sinus inflammation, 1 case of sphenoid sinus polyp, 1 case of skull base schwannoma, 1 case of inverted papilloma of sphenoid sinus, and 1 case of cerebrospinal fluid rhinorrhea.

**Interventions:**

In this study, 21 patients underwent endonasal endoscopic pterygoid sinus opening, and all symptoms improved after surgery.

**Outcomes:**

The specific type and clinical manifestations of 21 cases of isolated pterygoid sinus lesions were clarified by imaging and postoperative pathological analysis

**Lessons:**

Isolated pterygoid sinus lesions vary in type, and clinicians should combine clinical symptoms and imaging manifestations to improve diagnostic accuracy and reduce misdiagnosis. Systematic retrospective analysis helps to summarize diagnostic points and improve reference for clinical practice. Clinical symptoms and imaging examinations help to avoid misdiagnosis of this type of disease.

## 1. Introduction

The sphenoid sinus is located inside the sphenoid bone, behind and above the nasal cavity. It is adjacent to important structures such as the sella turcica, optic nerve, internal carotid artery, cavernous sinus, and the III, IV, V, VI pairs of cranial nerves.^[1]^ Among all sinuses, the anatomical position of the sphenoid sinus is the deepest and most concealed, and at the time of onset, it is limited to the sphenoid sinus cavity without typical clinical manifestations, making the diagnosis and treatment of diseases in the sphenoid sinus area difficult and leading to misdiagnosis and missed diagnosis. To further enhance understanding and avoid misdiagnosis, this study collected 21 patients with isolated sphenoid sinus lesions for retrospective analysis of their clinical manifestations, imaging findings, and postoperative pathological types, providing reference for the diagnosis and treatment of isolated sphenoid sinus lesions.

## 2. Clinical data

Search for clinical data of 21 patients with benign sphenoid sinus lesions treated in our department of otolaryngology from November 2021 to January 2023, including gender, age, clinical manifestations, imaging examinations, surgical methods, histopathology, etc. Inclusion criteria: imaging examination indicates isolated lesions of the sphenoid sinus (lesions do not involve the maxillary sinus, ethmoid sinus, or frontal sinus); received functional nasal endoscopic surgery at our hospital, and the postoperative diagnosis was confirmed by pathology; the patient has good compliance and complete follow-up data.

## 3. Results

Among the 21 cases, there were 7 male patients and 14 female patients. Among the initial symptoms, there were 12 cases of headache, 10 cases of nasal symptoms (including 9 cases of nasal congestion and 6 cases of runny nose), 2 cases of eye symptoms (1 case of blurred vision and 1 case of orbital pain), and 1 case of facial symptoms (1 case of involuntary facial twitching). Postoperative pathology revealed 10 cases (47.61%) of fungal sphenoid sinus inflammation, 3 cases (14.28%) of sphenoid sinus cyst, 4 cases (19.40%) of chronic sphenoid sinus inflammation, 1 case (4.76%) of sphenoid sinus polyp, 1 case (4.76%) of skull base schwannoma, 1 case (4.76%) of inverted papilloma of sphenoid sinus, and 1 case (4.76%) of cerebrospinal fluid rhinorrhea. The following are the basic information and symptoms of 21 patients (see Tables 1 and 2).

**Table 1:** The basic information of 21 patients.

Types of isolated pterygoid sinus lesions	Number of cases	Sex (male/female)	Average age in years/(zone)
Fungal sphenoid sinusitis	10	1/9	53.10 (20–74)
Parietal sinus cyst	3	0/3	43.00 (19–77)
Chronic inflammation of the mucous membranes	4	4/0	43.25 (14–72)
Butterfly sinus polyp	1	0/1	34.00 (34–34)
Inverted papilloma	1	0/1	70.00 (70–70)
Nerve sheath tumor	1	1/0	56.00 (56–56)
Cerebrospinal fluid (CSF) leakage from the pterygoid sinus	1	1/0	49.00 (49–49)

CSF = cerebrospinal fluid.

**Table 2:** The symptoms of 21 patients.

	Fungal sphenoid sinusitis	Parietal sinus cyst	Chronic inflammation of the mucous membranes	Butterfly sinus polyp	Inverted papilloma	Nerve sheath tumor	Cerebrospinal fluid (CSF) leakage from the pterygoid sinus
Asymptomatic	0	0	1	0	0	0	0
Headache	8	1	0	1	1	0	0
Stuffy nose	5	2	2	0	0	0	0
Sniffle	4	1	2	0	0	0	1
Blurred vision	0	0	1	0	0	0	0
Orbital pain	1	0	0	0	0	0	0
Facial symptoms	0	0	0	0	0	1	0

CSF = cerebrospinal fluid.

The following are the CT and MR imaging findings of 21 cases of isolated sphenoid sinus lesions (see Table 3).

**Table 3:** The CT and MRI imaging findings of 21 patients.

Types of isolated pterygoid sinus lesions	CT	MRI
Fungal sphenoid sinusitis	Sinus cavity of the pterygoid sinus is distended, with predominantly soft tissue density, mixed with multiple irregular high-density shadows, there may be bulging or resorption of the bone wall, sclerosis of the sinus wall bone, compressive resorption, thinning, and resorption of the bone in the adjacent saddle floor	Mixed signals are seen in the pterygoid sinus, inflammatory flaky exudative hyperdense shadows, fungal nodules with diminished T1 WI signals, short T1 signals with marked peripheral enhancement, and axial T2 WI with low signal shadows in the central region and long T2 signals in the periphery
Parietal sinus cyst	CT scan of the pterygoid sinus showed a homogeneous rounded fluid density shadow with well-defined borders, the cavity of the pterygoid sinus showed expansive growth and its bone resorption was thinning	T1 WI of the superior pterygoid sinus showed a spherical cystic occupation with long T1 signal shadow, regular linear enhancement of the cystic wall, and no calcification was seen, and axial T2 WI showed a long T2 irregular spherical signal shadow, and short T2 signal was reduced in the cystic fluid
Chronic inflammation of the mucous membranes	Enlargement of the pterygoid saddle, within which an isodense soft tissue shadow is seen, with compression and resorption of the surrounding bone	MRI-enhanced T1 scans show marked enhancement of the edematous mucosa of the pterygoid sinus, with no enhancement in the center of the lesion. MRI T2 W1 scans show long T2 signal shadows in the pterygoid sinus, with uneven signals due to the different protein contents
Butterfly sinus polyp	Soft tissue density shadows are seen in the pterygoid sinus, mucosal thickening is seen in the pterygoid sinus, and the wall of the pterygoid sinus is slightly irregular	MRI enhanced T1 high signal shadow, clear border, enhancement scan lesion, did not see obvious enhancement. T2 mixed slightly high signal shadow
Inverted papilloma	CT shows a soft tissue density mass in the right pterygoid sinus with homogeneous density and blurred sinus septum, which may show thinning, destruction or focal osteophytes of the affected bone wall under pressure	/
Nerve sheath tumor	CT shows a hypodense shadow of the left pterygoid sinus with well-defined and homogeneous features	A rounded mixed-signal nodular shadow is seen on the left side of the body of the pterygoid, with clear boundaries and a diameter of about 30 mm, within which multiple flow-vessel shadows are seen, and a few diffusion-restricted shadows are seen within it, with pushing changes in the surrounding tissues
Cerebrospinal fluid (CSF) leakage from the pterygoid sinus	CT showed soft tissue density shadows in the left pterygoid sinus, without Obvious bone defects were found	Continuous high signal shadow in the pterygoid sinus and intracranial cerebrospinal fluid phase is seen

CSF = cerebrospinal fluid, CT = computed tomography, MRI = magnetic resonance imaging.

### 3.1. Special cases

**Figure 1.**
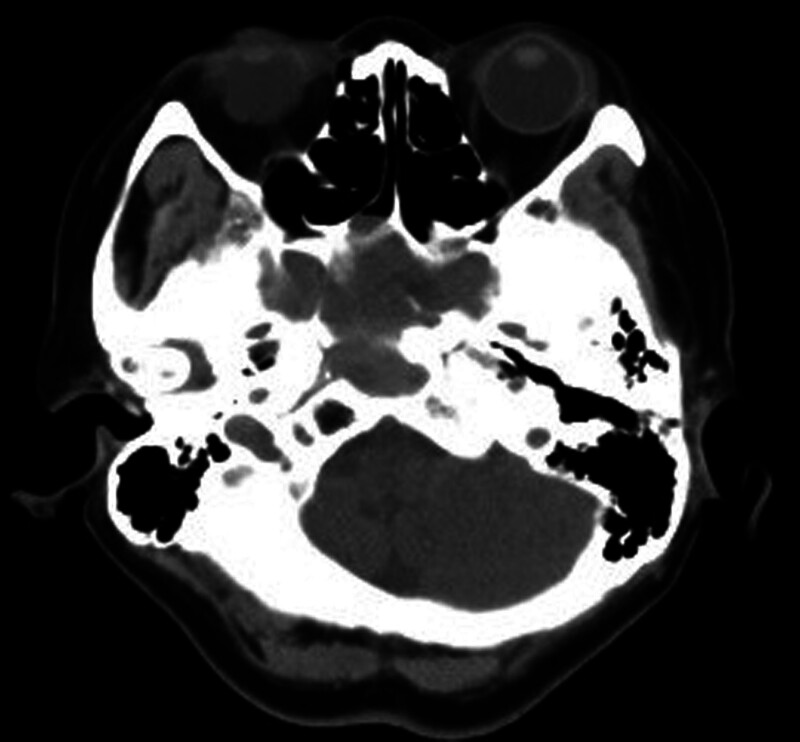
Patient, 70 years old, female, preoperative sinus CT reconstruction horizontal. CT = computed tomography.

**Figure 2.**
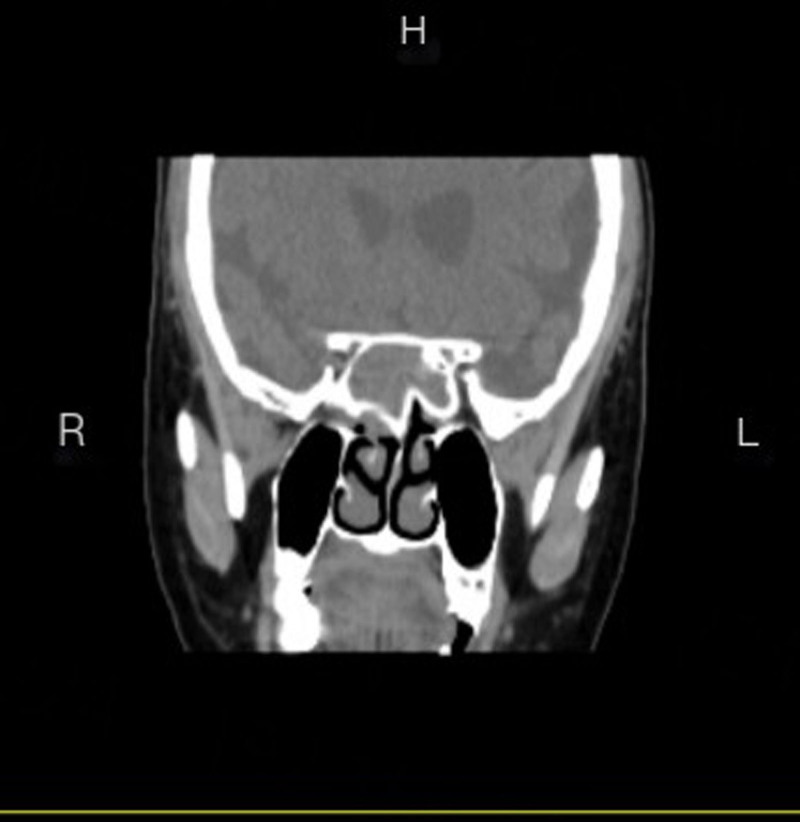
Patient, 70 years old, female, preoperative sinus CT reconstruction coronal. CT = computed tomography.

**Figure 3.**
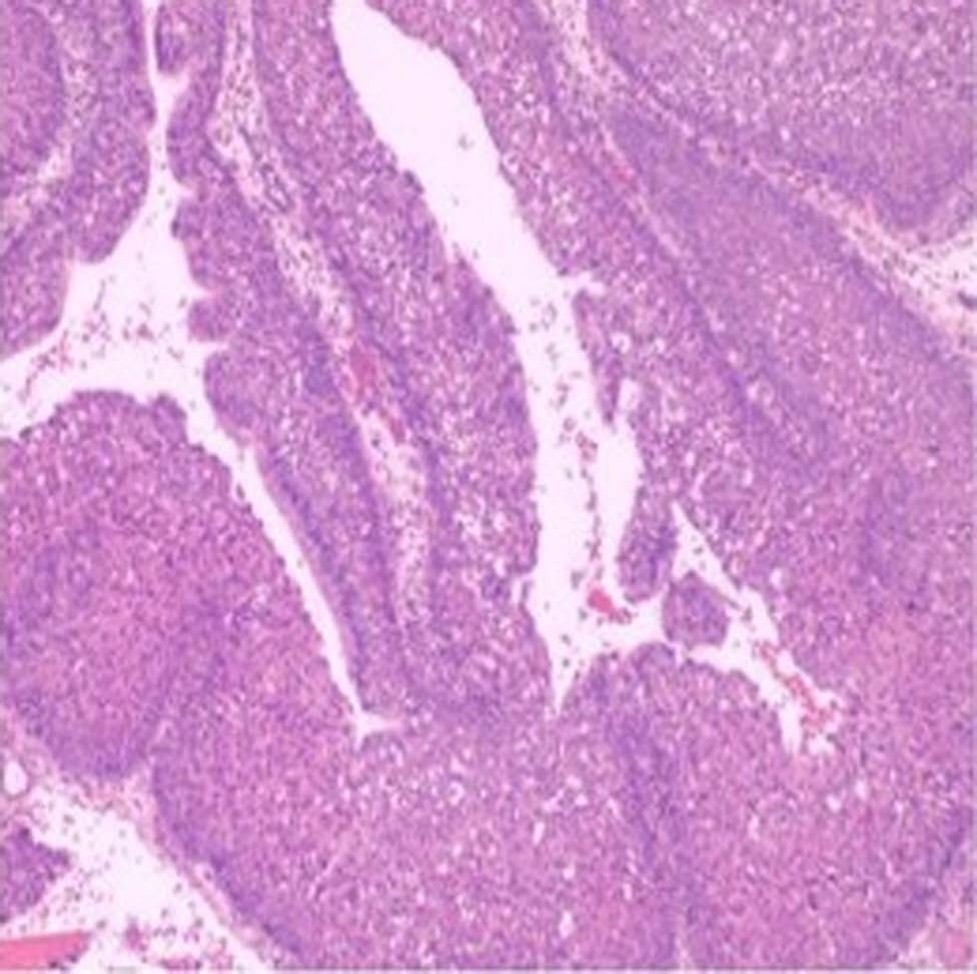
Patient, 70 years old, female histopathology.

Patient 1, female, 70 years old, was admitted to the hospital due to “recurrent right-sided headache for 1 year.” Specialized physical examination revealed a pale red new organism in the right sphenoid sinus recess. The CT of the sinus showed an enlarged sphenoid sinus filled with soft tissue density, thinning of the sinus wall, and no obvious signs of bone destruction (see Figs. 1 and 2). The surgical procedure involved “opening multiple sinuses on the right side under nasal endoscopy and removal of a mass in the right sphenoid sinus.” The postoperative pathology showed “inverted papilloma” (see Fig. 3).

**Figure 4.**
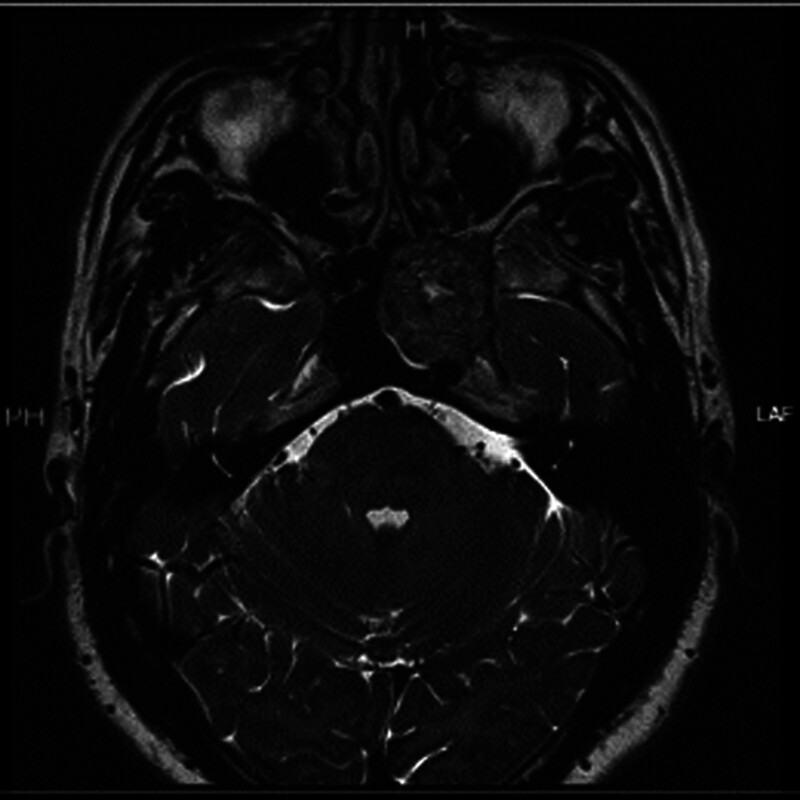
A 54-year old male patient with skull base schwannoma. Sinus MRI horizontal view. MRI = magnetic resonance imaging.

**Figure 5.**
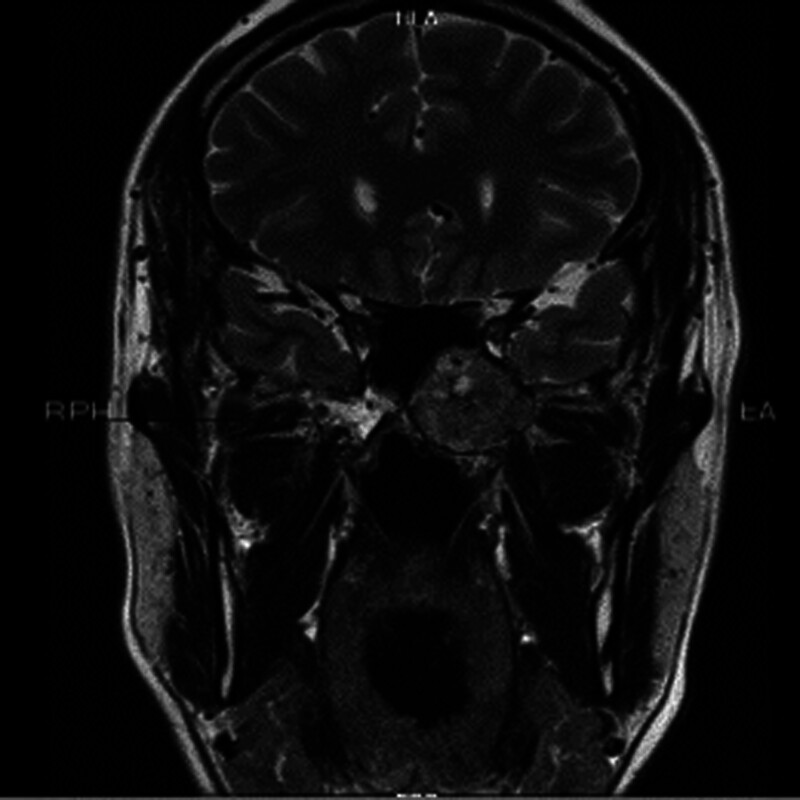
A 54-year old male patient with skull base schwannoma. Sinus MRI coronal view. MRI = magnetic resonance imaging.

**Figure 6.**
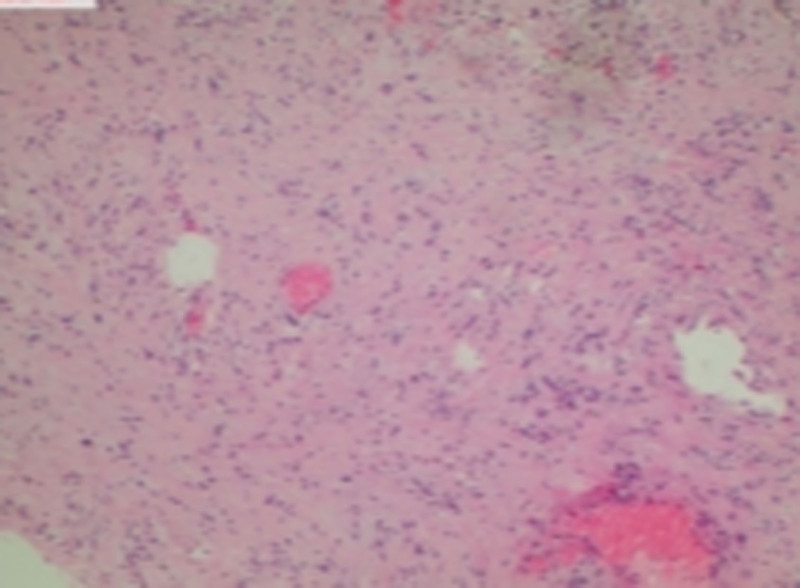
A 54-year old male patient with skull base schwannoma histopathology.

Patient 2, male, 54 years old, was admitted to the hospital due to “involuntary twitching of the left facial area for 2 years.” The cranial MRI showed: “A circular mixed signal nodule shadow can be seen on the left side of the sphenoid body, with clear boundaries and a diameter of about 30 mm. Multiple empty blood vessel shadows can be seen inside, and a few diffusion restricted shadows can be seen inside, with changes in surrounding tissue pushing” (see Figs. 4 and 5). The surgical method is “endoscopic transsphenoidal skull base tumor resection.” The postoperative pathology showed a “nerve sheath tumor” (see Fig. 6).

**Figure 7.**
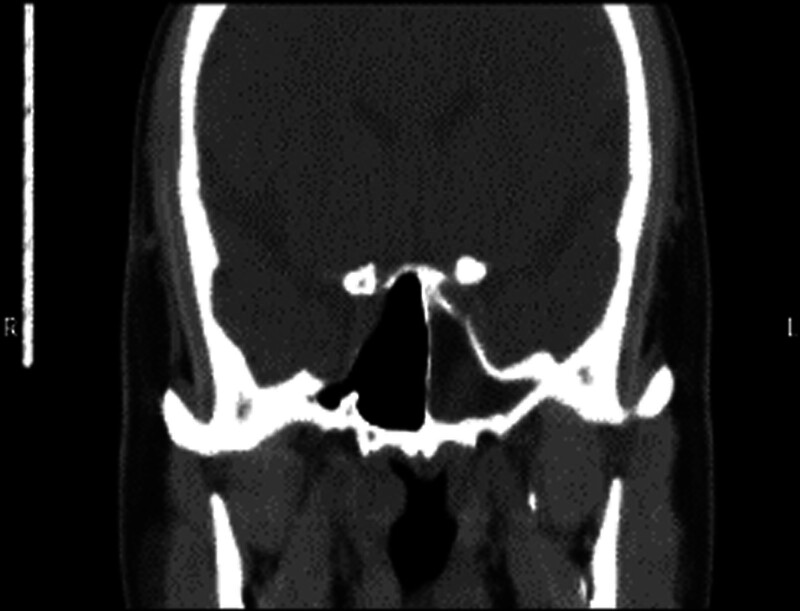
A 49-year old male patient with preoperative sinus CT coronal view. CT = computed tomography.

**Figure 8.**
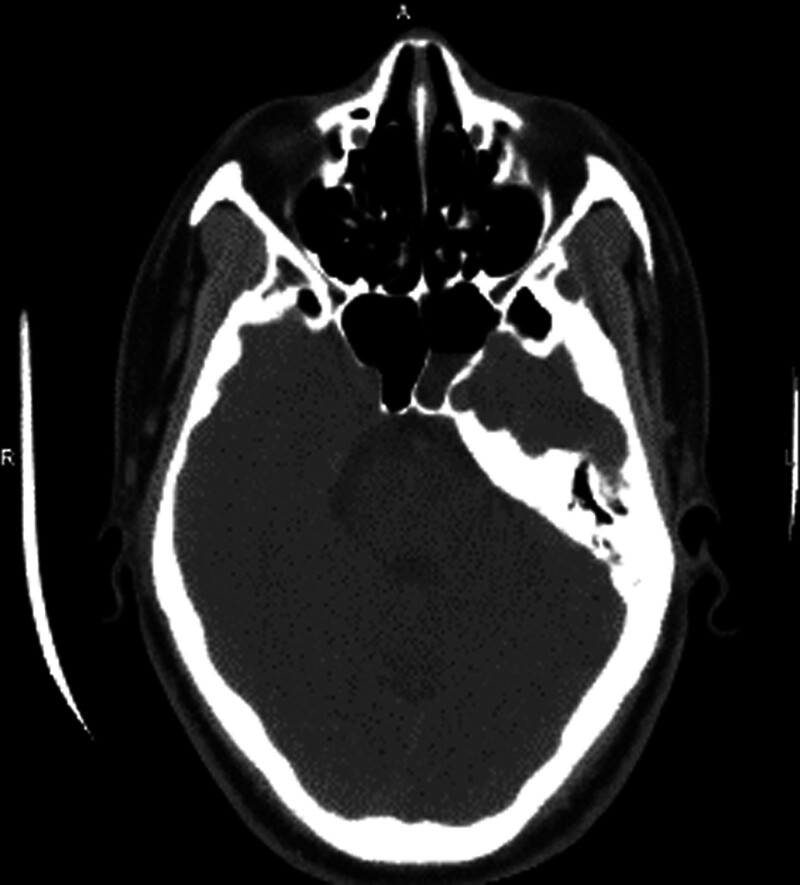
A 49-year old male patient with preoperative sinus CT horizontal position. CT = computed tomography.

**Figure 9.**
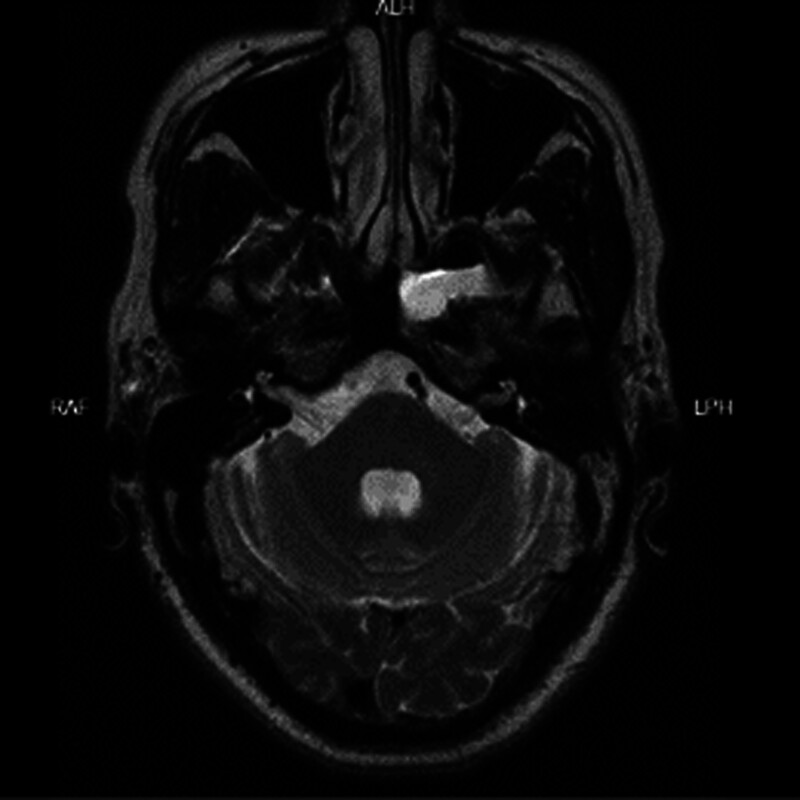
A 49-year old male patient with preoperative sinus MRI horizontal bitmap. MRI = magnetic resonance imaging.

**Figure 10.**
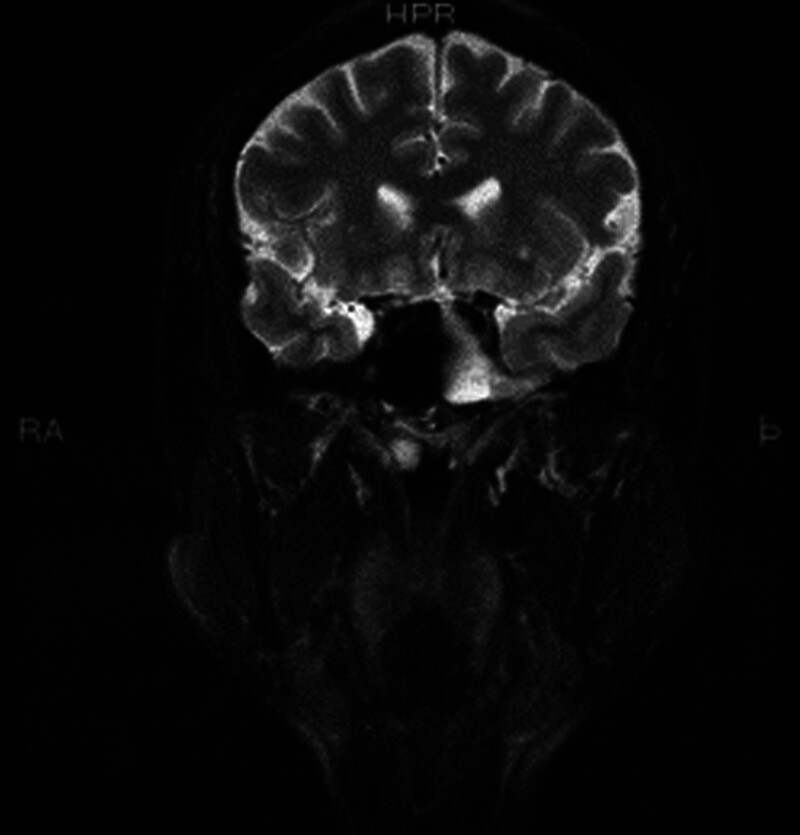
A 49-year old male patient with preoperative coronal map of sinus MRI. MRI = magnetic resonance imaging.

**Figure 11.**
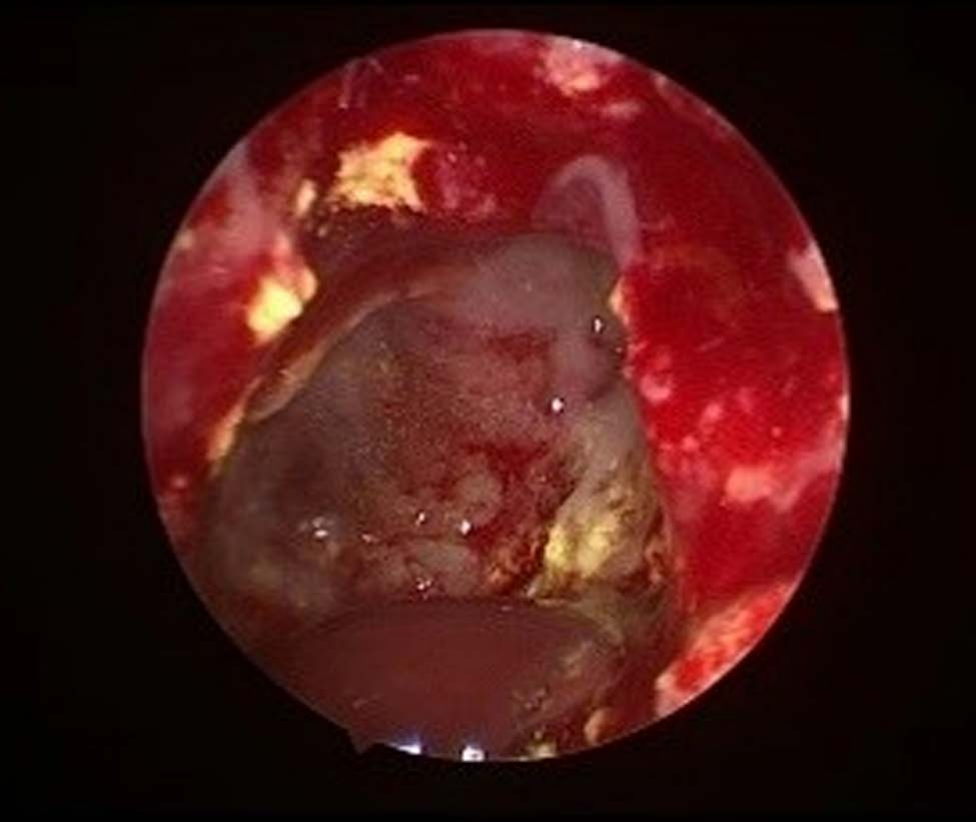
A 49-year old male patient intraoperative endoscopic visualization of the sphenoid sinus.

**Figure 12.**
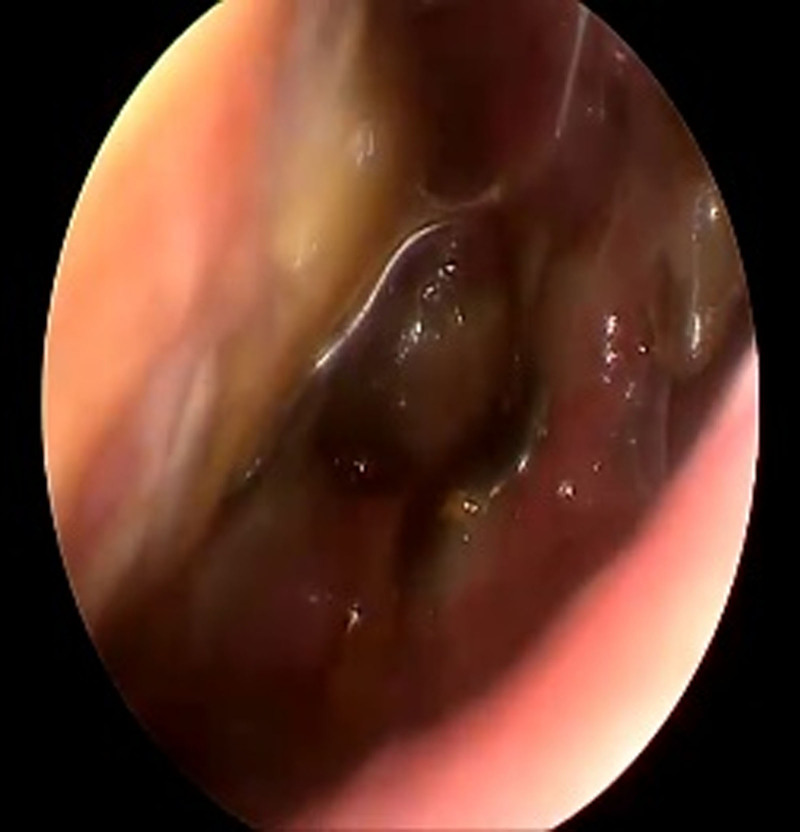
A 49-year old male patient 30 day postoperative reexamination of nasal endoscopy results.

Patient 3, male, 49 years old, was admitted to the hospital due to “repeated left nasal discharge for 2 years and recurrence for 2 months.” When looking down, clear colored liquid could be seen dripping from the left nasal cavity. Three dimensional CT of the sinus showed thickening of the mucosa in the right maxillary sinus, ethmoid sinus, and sphenoid sinus, with patchy high-density shadows in the maxillary sinus and ethmoid sinus cavities. The bony sinus walls were smooth, and the remaining sinus structures were clear. No abnormal density shadows were found in the sinus cavities. Sinus MRI showed that “the left sphenoid sinus was filled with long T1 and long T2 cerebrospinal fluid signal shadows, and the continuity of the local bone and mucosal surface on the top wall was interrupted. The local cerebrospinal fluid signal in the left middle cranial fossa was slightly higher than that on the right side” (see Figs. 7–10). The surgical method is “endoscopic repair of cerebrospinal fluid nasal fistula and left nasal sinus opening and pedicle composite tissue flap repair.” Intraoperative confirmation of cerebrospinal fluid rhinorrhea from the sphenoid sinus (Fig. 11). Figure 12 shows a photo of the endoscopic surgical area 1 month after surgery.

## 4. Discussions

A study reviewed published papers on isolated lesions of the sphenoid sinus collected over the past 25 years and found that the majority of lesions were non tumor lesions, with the most common being inflammatory lesions (50.3%), and fungal infections accounting for one-third of these infections. Next is mucinous cyst, accounting for 20.2%, while other lesions such as cerebrospinal fluid leakage, fibrous dysplasia, and inverted papilloma constitute the remaining part of the disease.^[2]^ The present study is consistent with this finding. The following text provides a detailed introduction to the symptoms.

### 4.1. Fungal sphenoid sinusitis

Among the 21 patients, fungal sphenoid sinusitis accounted for 10 cases, and the clinical manifestations were mostly unilateral, mainly headache, accompanied by nasal congestion, runny nose, or foul odor. The CT scan of the paranasal sinuses shows an expansion of the sphenoid sinus cavity, mainly composed of soft tissue density, mixed with multiple irregular high-density shadows. There may be bulging or absorption of the bone wall, hardening of the sinus wall bone, compressive absorption and thinning, and bone resorption near the sella floor.^[3]^ On the sinus MRI, mixed signals were observed in the sphenoid sinus, with inflammatory patchy exudate and high-density shadows. The signal of fungal nodules was weakened on T1 WI, showing short T1 signals and significantly enhanced in the periphery. On axial T2 WI, there was a low signal shadow in the central area and a long T2 signal shadow in the periphery, indicating mucosal edema and a fungal mass in the middle.

### 4.2. Pterygoid sinus cysts

Pterygoid sinus cysts are divided into mucosal cyst and mucinous cyst. The diagnosis of sphenoid sinus cyst mainly relies on imaging examination, and the consistency rate of CT and MRI examination is almost 100%.^[4]^ Among the 21 patients in this group, there were 3 patients with pathological type of sphenoid sinus cyst, and there were no misdiagnosed cases on imaging examination. CT scan shows a uniform circular liquid density shadow in the sphenoid sinus, with clear boundaries. The sphenoid sinus cavity shows expansive growth, and 2 (bone resorption thinning). MRI examination has high resolution of soft tissue, no bone artifacts, and can scan in any direction. Its ability to distinguish soft tissue and clarify the nature of lesions is significantly better than CT. On the axial T1 WI of sinus MRI, the sphenoid sinus shows a spherical cystic mass with long T1 signal shadow, regular linear enhancement of the cyst wall, long T2 irregular spherical signal shadow on the axial T2 WI, and reduced short T2 signal in the cyst fluid.^[5]^

### 4.3. Sphenoid sinus polyps

The main clinical manifestations of the patient are bilateral progressive nasal congestion, accompanied by clear or sticky nasal discharge. Some patients also have decreased sense of smell, and the head and face are stuffy and heavy. Among the 3 patients, one of them also had blurred vision. CT features: Soft tissue density shadow can be seen in the sphenoid sinus, mucosal thickening can be observed in the sphenoid sinus, and the sphenoid sinus wall is slightly irregular. MRI features: Enhanced scanning with T1 high signal shadow, 3 clear edges, enhanced scanning of lesions, no significant enhancement observed. T2 mixed with slightly higher signal intensity.

### 4.4. Inverted papilloma of the sphenoid sinus

Clinical manifestations are often unilateral, with persistent nasal congestion on 1 side of the nasal cavity, gradually worsening, accompanied by purulent nasal discharge, occasional bloody nasal discharge, occasional headaches, and olfactory abnormalities. The only case of inverted papilloma of the sphenoid sinus collected in this article was characterized by “recurrent right-sided headache for 1 year” as the main clinical presentation. CT showed a soft tissue density mass in the sphenoid sinus, with uniform density and blurred nasal (internasal) space. The affected bone wall may be compressed, thinned, damaged, or focal bone hyperplasia. Research has shown that CT scans are difficult to distinguish between inverted papilloma of the sinus and sinusitis, and the tumor outline is not clear enough, making it difficult to differentiate from other tumors. On the other hand, MRI has the advantages of no radiation, high soft tissue resolution, and multi plane imaging, which can clearly display signal changes in the internal structure of the tumor. The combination of CT and MRI in the diagnosis of inverted papilloma of the paranasal sinuses significantly improves the accuracy of staging diagnosis using CT or MRI alone, accurately determines the origin of the tumor, and provides favorable value for the diagnosis, staging, and surgical treatment of inverted papilloma of the paranasal sinuses The patient did not undergo MRI examination before surgery, and the diagnosis could not be confirmed before surgery.

### 4.5. Nerve sheath tumor

Among the 21 cases collected in this article, there was only 1 case of skull base schwannoma, and the source of the tumor was not correctly diagnosed before surgery. The patient was admitted due to “left facial twitching for 2 years.” MR imaging showed a circular mixed signal nodule shadow on the left side of the sphenoid body, with a clear boundary and a diameter of about 30 mm. Multiple empty blood vessel shadows were observed inside, and there were a few diffusion restricted shadows inside. The surrounding tissues were also affected. Although there is no significant specificity in the imaging findings of nasal and sinus schwannoma, preoperative imaging examination can help identify the extent and location of the tumor, which is of great significance for the selection of surgical methods. The main diagnosis of schwannoma depends on tissue biopsy, and specialized immunostaining can help with more accurate diagnosis.^[6]^

### 4.6. Cerebrospinal fluid nasal leakage from the pterygoid sinus

There was only 1 case of cerebrospinal fluid nasal leakage among the 21 cases collected in this paper. The main clinical manifestation of this patient is “intermittent or continuous outflow of cool watery fluid from the nasal cavity.” This case did not show any bone defects on CT, but on MRI, there were lines connecting the high signal shadows of cerebrospinal fluid and the high signal fluid shadows in the sinuses. The brain tissue and nasal mucosa showed obvious low signal. MR is more specific than CT in the diagnosis of cerebrospinal fluid rhinorrhea, and can also be diagnosed through physical signs and spinal (biological) examinations. The patient’s nasal endoscopy showed clear liquid coming out of the sphenoid sinus opening. The liquid was taken for examination and confirmed to be cerebrospinal fluid rhinorrhea. The preoperative diagnosis was clear.

The sphenoid sinus is located at the base of the skull and has complex relationships with important blood vessels, nerves, and other tissues, such as the internal carotid artery, optic nerve, pituitary gland, and cavernous sinus.^[7]^ In addition, simple sphenoid sinus inflammation does not show obvious symptoms in all 4 beds, and there are no characteristic clinical symptoms. It is difficult to obtain useful clinical clues through medical history inquiry and anterior nasal examination, and is easily overlooked in clinical practice. It is difficult to diagnose early and delay diagnosis and treatment, which will lead to serious complications.^[8]^ Due to the widespread application of CT, it lays the foundation for early diagnosis of isolated lesions of the sphenoid sinus. In previous studies, CT was considered the gold standard for diagnosing lesions of the sphenoid sinus. Its detection of bone and soft tissue has a very good contrast, while MR helps distinguish between inflammatory or tumorous soft tissue.^[9]^

There may be some possible limitations in this study. The small sample size of this paper, all from the same study center, may affect the generalizability and statistical validity of the results, and the uncertainty of the length of the patients’ postoperative follow-up, with the possibility of loss to follow-up, fails to adequately assess the long-term prognosis of the patients and the recurrence of the lesions. As a retrospective study, data collection and analysis relied on historical records, and there may be problems of undetailed records or inconsistent criteria.

This article aims to combine clinical symptoms and preoperative CT and MR to determine the nature of isolated sphenoid sinus lesions in 21 cases, provide more reference for surgical methods, reduce misdiagnosis, and have important guiding significance for the diagnosis and treatment of diseases.

## Acknowledgments

First of all, I would like to give my heartfelt thanks to all the people who have ever helped me in this paper. My sincere and hearty thanks and appreciations go firstly to my supervisor, Mr Chen Xudong, whose suggestions and encouragement have given me much insight into these translation studies. It has been a great privilege and joy to study under his guidance and supervision. Furthermore, it is my honor to benefit from his personality and diligence, which will treasure my whole life. My gratitude to him knows no bounds. I am also extremely grateful to all my friends and classmates who have kindly provided me assistance and companionship in the course of preparing this paper. In addition, many thanks go to my family for their unfailing love and unwavering support. Finally, I am really grateful to all those who devote much time to reading this thesis and give me much advice, which will benefit me in my later study.

## Author contributions

**Conceptualization:** Ziwei Yu.

**Data curation:** Ziwei Yu.

**Formal analysis:** Xudong Chen.

**Funding acquisition:** Xudong Chen.

**Methodology:** Yaowen Wang.

**Project administration:** Yanli Yu.

**Software:** Yanli Yu.

**Validation:** Yaowen Wang, Peng Cheng.
